# A Modern Technology Applied in Traditional Chinese Medicine: Progress and Future of the Nanotechnology in TCM

**DOI:** 10.1177/1559325819872854

**Published:** 2019-09-04

**Authors:** Guojun Yan, Yonglin Wang, Xinxin Han, Qian Zhang, Hui Xie, Jun Chen, De Ji, Chunqin Mao, Tulin Lu

**Affiliations:** 1 School of Pharmacy, Nanjing University of Chinese Medicine, Nanjing, Jiangsu, China; 2 Yangling Demonstration Zone Hospital, Xianyang, Shanxi, China

**Keywords:** nanotechnology, traditional Chinese medicine, preparation methods, nanocarriers

## Abstract

The application of nanotechnology to traditional Chinese medicine (TCM) enabled the development of Chinese medicine in the international society. The pharmacodynamics of TCM is not only depending on its chemical constituents but also related to its physical state such as particle size. Indeed, there is some new pesticide effect that appeared when the medicine was being made into nanophase. The application of nanotechnology to TCM can expand the use of a range of Chinese medicinal materials. In this review, we introduce the concept of nanometer TCM. We also review the preparation methods, advantages, and development tendency of Nano-TCM; furthermore, we analyze the problems in the process of development of Nano-TCM and put forward varies possible solutions to solve this problems, thereby providing new thought for the development of Nano-TCM.

## Introduction

Nanotechnology is the science of engineering materials and systems on a molecular scale. Its application to medicine, nanomedicine, has enabled the development of nanoparticle drug-delivery vehicles. These nanocarriers are generally <100 nm in size and have the ability to carry and deliver therapeutics to disease sites.^1-5^ It is a new technology that was born and grew in 1980s and is taken for the source of various emerging technologies of 21st century. Furthermore, nanotechnology, biotechnology, and information technology are considered the 3 key technologies of 21st century. With putting forward “nano-traditional Chinese medicine (TCM)” in 1988, the research of nanoparticles technology in the field of TCM has been paid more and more attention. Nano-TCM is the application of nanotechnology to manufacture the effective components, effective parts, original medicine, and compound preparation of TCM; it is the product of TCM nanocrystallization rather than a new kind of medicine. When TCM are produced in nanoscale, its physical specification, chemical properties, and biological characteristic will have a great change and generate new pharmacodynamics. Compared to native TCM, nano-TCM not only increases the bioavailability of medicine and strengthens the targeted effect but also reduces adverse reactions that provide a new research method for the further development of modernization of TCM.^6,7^

## Preparation of Nano-TCM

There are wide varieties of Chinese medicine; hence, different kinds of Chinese medicine need use different processing methods; this principle is due to the characteristics of Chinese medicine. Mineral medicines usually are very hard, and the traditional processing method is calcining and quenching; the herb medicines are rich in fiber and are usually very tenacious and difficult to crush, so extracting with water was always used in traditional processing. The current method is direct nanocrystallization of the TCM or carrying drugs using nanocarrier, base on it, and blend it in right amount in modern preparation technology.

### Chinese Herbal Medicine Nanotechnology

The surface area of Chinese herbal medicine particles will be expanded after super fine crushing which will provide a number of activated atoms, thereby making the medicine accrue plenty of physicochemical properties and biological activity that does not exist under normal condition. This will shorten the time of decoction, improve the solubility and dissolution rate, and strengthen the effect. For some medicine with single ingredient and effect such as mineral medicine, or some medicine with special activity, it is feasible to apply ultra-fine pulverization technology instead of conventional crushing method.^6^

### Mechanical Comminuting Process

**Figure 1.**
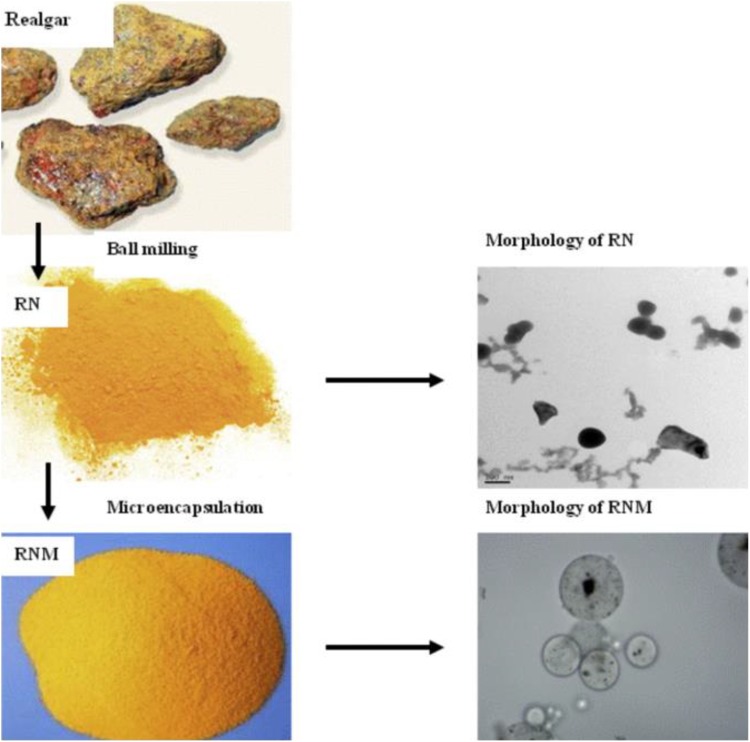
Operation flowchart for preparation of the realgar unit. RN indicates realgar nanoparticles; RNM, RN-based microcapsules. (Order through Copyright Clearance Center’s RightsLink service, Order Number: 4574070838606)

Mechanical pulverization is the process of making massive solid matter crushed into specified fineness by means of mechanical force. It is the major method for micronized treatment of solid drugs, including ball-milling method and airflow smash method.^6^ In Shi et al’s research,^8^ they use nanotechnology to process Niuhuang Xingxiao Wan (NXW). NXW was formulated into 4 units, that is, realgar, frankincense and myrrh oil (FMO), musk, and bezoars. The realgar was processed using the wet ball milling (Figure 1). After formulation, the 4 independent units prepared were encapsulated together to obtain the final Niuhuang Xingxiao Wan-multi-unit drug delivery system (NXW-MUDDS). Pharmacokinetic studies showed that the area under the plasma concentration–time curve (AUC), terminal half-life (T-1/2), and time to reach the peak plasma concentration (T-max) following administration of NXW-MUDDS were 5.21, 1.96, and 1.99 times higher, respectively, than that of NXW. The in vivo antitumor activity assay showed that the efficacy of NXW-MUDDS was significantly higher (*P* < .05) than that of NXW. Collectively, these results demonstrate that wet ball milling can be used in the pharmaceutical processing of TCM. From Karthik et al’s article,^9^ the herbal nanoparticles were prepared from shade-dried *Tridax procumbens* plant leaves using ball milling technique with different process parameters, such as ball ratio/size and milling time. They controlled the nanoparticle size by controlling the process of ball milling. The increase in ball ratio and milling time periods leads to a decrease in nanoparticle size from 114 to 45 nm which in turn increases the antimicrobial activities. It illustrates that nanoparticle size can influence antimicrobial activity, thereby indicating ball milling can be applied to produce nano-TCM.

### High-Pressure Microfluidization

**Figure 2.**
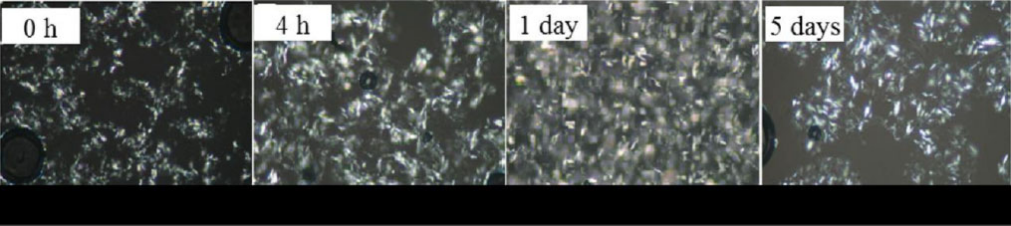
Grayscale polarized light microscopy (PLM) images of palm stearin/palm olein (PS/PO) oil control blend crystal networks stored for various periods: 0 hour (onset of storage), 4 hours, 1 day, and 5 days. Magnification 500 × 19.

**Figure 3. fig3-1559325819872854:**
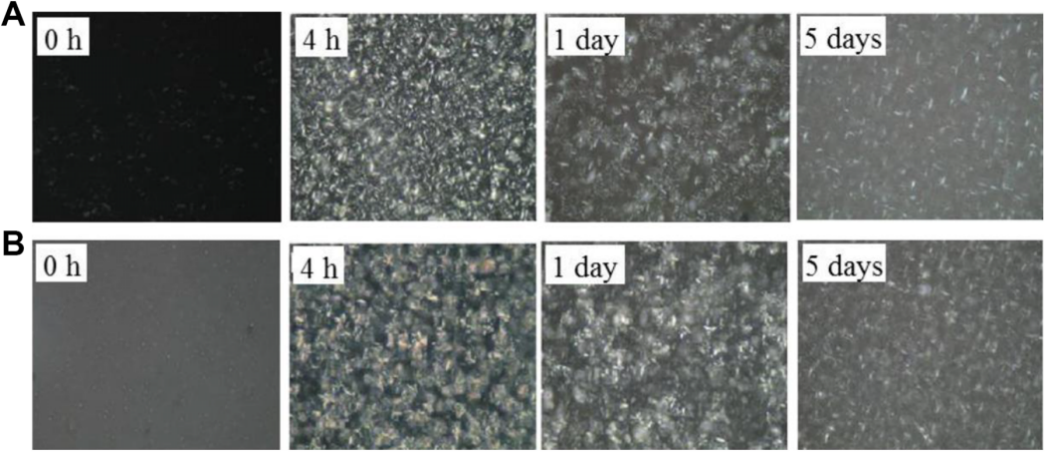
Polarized light photomicrographs for the PS/PO oil blends under 60 MPa high-pressure microfluidization (HPM) treatment. A, Treated once and crystallized 0 hour, 4 hours, 1 day, and 5 days. B, Treated twice and crystallized 0 hour, 4 hours, 1 day, and 5 days, respectively. Magnification 500 × 19.

High-pressure microfluidization (HPM) is a new technology that employs a device called a microfluidizer. This device uses a high-pressure positive displacement pump (the pressure range is approximately 5-200 MPa). This equipment has been traditionally used in the pharmaceutical industry to make pharmaceutical emulsions as well as in the food industry to produce nanosystems^10,11^ or homogenized proteins (milk, whey protein, trypsin, and so on)^12-15^ and dietary fiber^16^ only in the last few years. High-pressure microfluidization uses the combined forces of high-velocity impact, high-frequency vibration, instantaneous pressure drop, intense shear, cavitation, and ultra-high pressures up to 200 MPa with a short treatment time (less than 5 seconds) and continuous operation.^17,18^ In Han et al’s study,^19^ they performed research on effect of HPM on the crystallization behavior of palm stearin. In order to find the relationship, they used moderate and high microfluidization pressures (60 and 120 MPa) and different treatment times (once and twice). Eventually, they found that HPM treatment was more likely to modify the crystallization processes and nucleation mechanisms (Figures 2 [Fig fig3-1559325819872854]–4).

**Figure 4.**
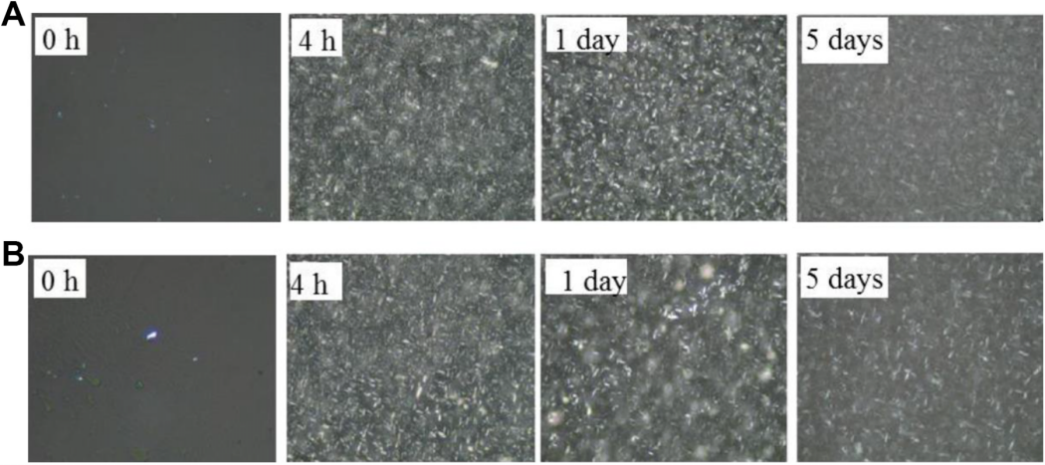
Polarized light photomicrographs for the PS/PO oil blends under 120 MPa high-pressure microfluidization (HPM) treatment. A, Treated once and crystallized 0 hour, 4 hours, 1 day, and 5 days. B, Treated twice and crystallized 0 hour, 4 hours, 1 day, and 5 days, respectively. Magnification 500 × 19.

### Microwave Technology

Microwave technology is a kind of technology that can penetrate into any substance. Moreover, the heated level depends on the dielectric properties. So, the microwave technology provides a new way of heating and drying in the field of pharmaceutics.^20^ Cao et al^21^ use microwave-assisted aggregation-induced emission (AIE) to make active fluorescent polymeric nanoparticles (FPNs). The result showed that polyethylene glycol methacrylate-thermoplastic elastomer (PEGMA-TPE) FPNs are prepared through microwave-assisted Kabachnik-Fields reaction.

### Nanocarrier

Nanocarrier technology is a method that uses nanomaterial for carrying drugs as carrier materials. Currently, there are many kinds of technologies including solid dispersion, inclusion technology, polymer nanoparticles carrier technology, and super emulsify nanometer dispersion technology.^22-27^

### Inclusion Technology

The carrier material used by inclusion technology is a kind of nanoscale molecular material, mainly cyclodextrin (CD) that maintains 3 kinds, α, β, γ and their ramification. These 3 CDs are all tubular in structure, which can reduce the drug’s irritation and increase its stability. In Fan et al’s article,^28^ they showed an example that can solve the problem of ellagic acid which is difficult to dissolve and absorb and increase the time of pesticide effect. Under alkaline conditions, Ellagic acid is dissolved in water in an ionic state, and the inclusion CD is added into the mixture for 10 to 120 minutes for acidification to obtain the ellagic acid–CD precipitate. After the ultrasonic fusion reaction, made of colloidal solution prepared by spray-drying white powder formulations, ellagic acid CD inclusion complex sustained release agent. This provides a good basis for preparation of nano-Chinese medicine inclusion carrier.^29^

### Polymer Nanoparticles Carrier Technology

Polymer nanoparticles usually have 2 types of preparation methods: The first is based on good biocompatibility and biodegradability of aliphatic polyester-based materials and poly amino acids as the carrier; the second method is the use of amphiphilic polymerization micelles, that is, some polymers with hydrophobic and hydrophilic segments form micelles under certain conditions to carry hydrophobic drugs and to protect the specific activity of certain components of TCM.^30^ The combination of the drug and the polymer nanoparticle can be encapsulated or can be attached or grafted. The modification of the polymer particle surface can also improve the performance of the nanoparticle. At present, the combination of the magnetic particle and the carrier material is mostly used to increase the drug target sexuality.

### Liposome Nanoparticles Carrier Technology

Liposome is a kind of closed vesicle with structure similar to biomembrane that is the most convenient and safety temperature-controlled release drug carrier. Liposome is composed of phospholipids and cholesterol, and cholesterol has effect on the liposome physiochemical properties.^31^ Currently, the preparation method of nanoliposome are solvent evaporation method, thin film evaporation method, freeze-drying method, and ultrasonic dispersion method. Yang and Qian^32^ used solvent evaporation method to prepare tetramethrin microcapsule; as a result, tetramethrin microcapsule with uniform size, regular shape, and complete structure was successfully prepared with 1.2% of ethyl cellulose, 35% of methylene chloride, and 4% of emulsifier R. The average particle size was 56.8 μm, and the encapsulation efficiency of microcapsule was 50.7%. Compared to primary medicine, tetramethrin microcapsule has significant sustained-release properties. In Pu et al’s research,^33^ they prepared thymol liposome using film evaporation method and compared to ethanol injection method; the result shows that the particle size of liposomes prepared by thin film evaporation method was comparable to that of ethanol injection method, but the dispersion and encapsulation efficiencies were better than that of the latter.

### Solid Dispersion Technology

Solid dispersion technology is the one where the slightly soluble solid drug is made to disperse into another water soluble material or slightly soluble, enteric soluble material with molecule in colloidal, microcrystal, or amorphous state. After dispersion, the drug usually exists in a microcrystal, micro emulsion, or molecule state. It is a kind of hyperdispersion system that has quick acting and high efficiency characteristics.^34,35^ In Ogawa et al’s^36^ research, they found that solid dispersion technology could improve the water solubility of drugs. It prepared solid dispersion particles using hot-melt extrusion and spray drying. Indomethacin (IMC) was used as a model comprising drugs with low solubility in water, and d-mannitol was used as an excipient. As a result, they concluded that dissolution behavior of the original drug crystal could be improved by solid dispersion with the polyvinyl caprolactam-polyvinyl acetate-polyethylene glycol graft copolymer. The powder X-ray diffraction pattern and thermal analysis indicated that the solid dispersion prepared with the polyvinyl caprolactam-polyvinyl acetate-polyethylene glycol graft copolymer and IMC was in an amorphous state. In the meantime, Bipin et al also did some research on it. Cinnarizine was prepared with a polyethylene glycol 4000 and polyvinyl pyrrolidone K30 using solvent evaporation and fusion method in the 1:1, 1:2, and 1:3 ratio of drug and carrier, respectively, and evaluated it by using infrared spectroscopy and in vitro dissolution study. The results showed that the dissolution rate of polyethylene glycol and polyvinyl pyrrolidone was increased.^37^

## The Advantages of Nano-TCM

Nano-Chinese medicine is used not only to smash the drug to the nanoscale but also the composition of the prescription of the effective part of the drug or active ingredients through nanotechnology processing, giving TCM to new functions. Nano-Chinese medicine has the following characteristics.

### Improving Bioavailability

In Nano-Chinese medicine, the cell wall has been broken, so the active ingredient is more easily absorbed by the body. In addition, after nanocrystallization, surface area of the Chinese medicine will increase, the contacting area with the interface will increase, and so it will more easily to dissolve. Furthermore, due to the increase in specific surface area, the contact area of the drug and dose part will increase, which will lengthen the residence time of the drug in the body and significantly increase the absorption amount of the drug.^38^ In Ren research,^39^ a contrast is made between albendazole (ABZ) raw material and albendazole nanopowder in bioavailabillity by performing an animal experiment. Their group initially established a method for the determination of ABZ and its metabolites in biological samples by high-performance liquid chromatography. The plasma concentrations of ABZ and its major metabolites in rat plasma were determined and treated with 3p97 pharmacokinetic software to investigate the effect of drug on the bioavailability of ABZ after nanocrystallization. The result indicated that the ABZ nanopowder in rats is consistent with 2-compartment pharmacokinetic model. Compared to the raw material of ABZ, the relative bioavailability of ABZ nanopowder is 157.03%; hence, nanocrystallization of medicine can improve absorption rate of the drug, increase absorption of the drug, and improve oral bioavailability of ABZ.^39^

### Lowering the Toxicity of Drug

Drugs enter the body through a variety of ways to trigger a wide range of toxic effects at different levels. Many of these toxicities are related to free radicals and oxidative damage. Under physiological conditions, there is a dynamic balance between production and clearance of free radicals in the body. However, under certain pathological conditions, free radicals are generated excessively, which can cause tissue damage beyond the capacity of clearance. In Ling and Shen article,^40^ in clinical use, cisplatin has promising effects but is limited by its systemic side effects; therefore, they synthesize a stimuli-sensitive Pt (IV) prodrug and tether to ethylenediamine-modified hyaluronic acid to form a tumor-targeting hyaluronic acid-ethylenediamine-platinum (IV) (HA-EDA-Pt(IV)) nanoconjugate with reduced adverse reactions and enhanced efficacy. The results indicated that the nanomedicine significantly alleviated toxic side effects.^41^

### Enhancing the Pesticide Effect and Increasing New Drug Effect

Ling et al^42^ have done a series of experiments to indicate that herpetrione nanosuspension (PEDX-NS) exhibits anti-hepatitis B virus (HBV) activity both in vitro and in vivo and its effect was superior to that of herpetrione coarse suspension (PEDX-CS). In this experiment, it adopted 2.2.15 cell model and duck HBV infection model to study antiviral activity of PEDX-NS in vitro and in vivo and compared with PEDX-CS.

### Targeting Effect

Currently, brain-targeted nanocarrier systems are becoming study hotspot. The blood–brain barrier (BBB) is considered to be the major obstacle in treating central nervous system diseases. The BBB protects brain from harmful substances and maintains the steady state of microenvironment of brain. Meanwhile, BBB also gives rise to the poor penetration of drugs into brain. With the development of nanotechnology, the nanocarriers such as liposomes, nanoparticles, micelles, nanogel, microemulsion, and solid lipid nanoparticles for delivering drugs make it possible to transport drugs across the BBB. Therefore, possible strategies for brain targeting could be available.^43^ Yin et al^44^ prepared a kind of novel nanoparticles of carboxymethyl chitosan covered with C-phycocyanin carrying CD59sp and evaluated its killing effect on HeLa cells. Eventually, they concluded that the novel targeting C-phycocyanin (CPC) nanoparticles could inhibit growth of HeLa cell, with superior apoptosis inducing effect over the other drugs, which provided a new idea for the research on marine drugs and an important theoretical value for further study on targeting drug-loading nanoparticles.

### Sustained and Controlled Release

With polymer nanoparticles as a carrier and other technical means, the drug can achieve sustained release and controlled release.^45^ Sun et al^46^ did some research on sustained-release behavior of the ofloxacin/montmorillonoid (OFLO/MMT) nanocomposite. They used the solution ion exchange to prepare the OFLO/MMT nanocomposite and used single-factor method to study drug initial concentration, reaction time, and the reaction temperature on the drug loading of the nanocomposite. The results showed that the sustained-release behavior was obviously observed in in vitro release experiment for the OFLO/MMT composite, and the release kinetic behavior in acid medium was different from that of basic medium. There are few studies on sustained and controlled release on TCM.

### Rich Dosage Form Available

The use of nanotechnology micronized drug can be applied to oral controlled-release tablets, buccal tablets, sprays, instant oral tablets and liposomes, and other dosage forms.^6^ Dong et al^47^ used high-gravity antisolvent to prepare a novel site-specific drug delivery system that can improve bioavailability and drug efficacy.

## Development of Nano-TCM

As the most innovative innovation in the modernization of Chinese medicine, nano-Chinese medicine holds an infinite prospect and huge potential for industrial expansion. Although the development of nanotechnology is booming, the nanotechnology of TCM is still in its infancy. How to correctly and reasonably apply nanotechnology to the study of TCM is a question worth pondering.^6^

### Combine With TCM Theory

The pharmacologic action of Chinese herbology is considerably complex, and it needs to combine TCM theory with nanotechnology, with the guidance of TCM-theory, guaranteeing Chinese medicine rich ingredient, target spot, and methods.

### The Preparation Problems of Nano-TCM

Currently, the research about the preparation of nano-TCM is still imperfect, and there are a number of problems to be settled urgently. For example, after nanomaterialization, the medicine will change its active ingredient and physicochemical properties, and it may destroy the effective constituent of drug. When the TCM are prepared as nanomedicine, it may correct its defect. On the contrary, it can also increase the toxicity.^6^

### Problems of Stability

After nanogrinding, surface area of Chinese medicine will be increased, making the drug particles to easily unite, provide more stability, will reduce difficulty to store, and the active ingredient and pharmacodynamics of uncertainty will give the drug quality and stability of the controllable left hidden problems. How to improve the stability of nanomedicine is also a problem that has to be solved.^6^

## Future Directions

As a newly emerging science and technology with great market potential, nanotechnology will have far-reaching impact on humans. It will become one of the leading new technologies in the 21st century. Traditional Chinese medicine theory is a treasure of Chinese excellent culture and has a history of thousands of years. However, due to the backward comprehensive technology in the field of TCM, it is urgent to apply new technologies and new methods to TCM, thereby introducing nanotechnology into modern Chinese medicine can improve it. It is of great significance to solve the problems of low bioavailability, lack of reliable quality standards, and single dosage form of the conventional preparations of TCM by researching and developing new nano-dosage forms and utilizing the advantages of the nano-drug loading system.^6^
